# Identification of immunity-related genes in *Plutella xylostella* in response to fungal peptide destruxin A: RNA-Seq and DGE analysis

**DOI:** 10.1038/s41598-017-11298-7

**Published:** 2017-09-08

**Authors:** Muhammad Shakeel, Xiaoxia Xu, Jin Xu, Xun Zhu, Shuzhong Li, Xianqiang Zhou, Jialin Yu, Xiaojing Xu, Qiongbo Hu, Xiaoqiang Yu, Fengliang Jin

**Affiliations:** 1 College of Agriculture, South China Agricultural University, Laboratory of Bio-Pesticide Creation and Application of Guangdong Province, Guangzhou, P.R. China; 2 State Key Laboratory for Biology of Plant Disease and Insect Pests, Institute of Plant Protection, Chinese Academy of Agricultural Sciences, Beijing, 100193 China; 3 BGI-Shenzhen, Shenzhen, P.R. China; 4 School of Biological Sciences, University of Missouri-Kansas, Kansas City, MO 64110 USA

## Abstract

*Plutella xylostella* has become the major lepidopteran pest of *Brassica* owing to its strong ability of resistance development to a wide range of insecticides. Destruxin A, a mycotoxin of entomopathogenic fungus, *Metarhizium anisopliae*, has broad-spectrum insecticidal effects. However, the interaction mechanism of destruxin A with the immune system of *P. xylostella* at genomic level is still not well understood. Here, we identified 129 immunity-related genes, including pattern recognition receptors, signal modulators, few members of main immune pathways (Toll, Imd, and JAK/STAT), and immune effectors in *P. xylostella* in response to destruxin A at three different time courses (2 h, 4 h, and 6 h). It is worthy to mention that the immunity-related differentially expressed genes (DEGs) analysis exhibited 30, 78, and 72 up-regulated and 17, 13, and 6 down-regulated genes in *P. xylostella* after destruxin A injection at 2 h, 4 h, and 6 h, respectively, compared to control. Interestingly, our results revealed that the expression of antimicrobial peptides that play a vital role in insect immune system was up-regulated after the injection of destruxin A. Our findings provide a detailed information on immunity-related DEGs and reveal the potential of *P. xylostella* to limit the infection of fungal peptide destruxin A by increasing the activity of antimicrobial peptides.

## Introduction

The diamondback moth, *Plutella xylostella*, has become the major lepidopteran pest of *Brassica* worldwide in the past four decades costing approximately US$4 billion annually on its management^1^. The attributes like high reproductive potential, lack of natural enemies, and its strong ability of resistance development to a wide range of insecticides and growth regulators, are the reasons for its continued success against modern pest management approaches^2^. Until now, *P. xylostella* has evolved resistance to almost all classes of insecticides and *Bacillus thuringiensis*-based products^2, 3^. At present, there is a need to develop novel biological control methods, to reduce harmful effects of insecticides, as alternative control strategies^4^.

The entomopathogenic fungi, such as *Metarhizium anisopliae* and *Beauveria bassiana*, are widely considered as important biological control agents^5–7^, and *M. anisopliae* has commercially been used for controlling insect pests^8–11^. The reason for successfully infecting a wide range of insects could be secretion of virulence factors by some fungi during pathogenesis. Destruxins, the secondary metabolites of fungi, produced by entomopathogenic fungi like *M. anisopliae* and *Aschersonia* spp. are considered as vital virulence factors accelerating the death of insects^12–14^.

Chemically, destruxins have a typical composition containing α-hydroxy acid and five amino acids which form cyclic hexadepsipeptides. Until now, 39 analogs of destruxins have been extracted from various fungal species^15–17^. Among them, few destruxins such as Destruxin A, Destruxin B, and Destruxin E have exhibited significant insecticidal activities against various insect pests^12, 18, 19^. Previously, it has been shown that destruxins inhibit V-type ATPase hydrolytic activity of *Galleria mellonella*, prompt oxidative stress in *Spodoptera litura* and affect the Ca^2+^ channel in muscle cells of *Manduca sexta* ^20–22^. Additionally, destruxins are also reported to affect the immune system of insects, such as *Drosophila melanogaster* innate immune response was suppressed by destruxin A following the inhibition of antimicrobial peptides^23^, however, no significant changes in the expression of antimicrobial peptides were observed in hemocytes of *Bombyx mori* in response to destruxin A^24^.

Invertebrates, unlike mammals, don’t have an adaptive immune system, but instead, they rely on a sophisticated innate immune system for defense against invading microbes. The innate immune system of insects is comprised of two main components, cellular and humoral immune responses^25^. The former relies majorly on the action of hemocytes in the phagocytosis of pathogens^26^, while the latter refers to the process of melanization with phenoloxidases^27^ and synthesis of immune effector molecules^28^.

To date, with the help of genome-wide analysis, immunity-related genes and gene families have been identified in various insect species including *P. xylostella* ^29–32^. Prior to the genome sequence of *P. xylostella*, immunity-related genes were identified by using expressed sequence tags and cDNA microarray analysis^33^, however, recently Xia *et al*.^32^ reanalyzed the immunity-related genes of *P. xylostella* in response to bacterial infection, to better understand the mechanism of immunity-related genes, at the genomic level. Similarly, Etebari *et al*.^34^ identified microRNAs from *P. xylostella* in response to parasitization by *Diadegma semiclausum* using the genome of other lepidopteran species as proxy references. Recently, Etebari *et al*.^35^ also revised the annotation of microRNAs, as the use of other species genomes as proxy references may cause errors and the level of errors is unknown^35^. Previously, Han *et al*.^36^ compared the expression pattern of gene profiles of *P. xylostella* between control and destruxin A treatment only at one-time point (4 h) at the larval stage by using transcriptome of *P. xylostella* as a background. Keeping in view the importance of an availability of genome sequence, in the present study, we also reanalyzed the expression pattern of immunity-related genes of *P. xylostella* by comparing control with destruxin A at the genomic level. The comparison of control with only one treatment performed by Han *et al*.^36^ seemed to be insufficient to show dynamical changes of differentially expressed genes (DEGs) in response to destruxin A, as the gene expression profiling of different time points can provide DEGs dynamical behavior information, thus, we compared control with destruxin A at three-time courses (2 h, 4 h, and 6 h) of the larval stage at genomic level by using RNA-Seq and DGE methods. Our results will not only provide deep insight into immunogenetics of *P. xylostella* in response to destruxin A, but will also improve the current understanding of host-pathogen interactions at the genomic level.

## Results

### Summary of Illumina sequencing and gene assembly

To acquire detailed information about the genes and their networks which control the immune system of *P. xylostella* in response to pathogens, especially fungal secondary metabolite destruxin A, at the genomic level, the 4^th^ larval instar was injected with destruxin A. Four cDNA libraries were generated from control and destruxin A treated at different time courses (2 h, 4 h, and 6 h), and then sequenced using Illumina HiSeq^TM^ 2000 system. After filtering out the adapter sequences and low-quality reads, the Q20, Q30, and GC percentages of the samples were as follows: (1) control, 98.2, 94.6, and 47.8%; (2) 2 h, 98.8, 96.7, and 49.52%; (3) 4 h, 98.2, 94.4, and 47.49%, and (4) 6 h, 98.9, 96.8, and 48.75%, respectively (Supplementary Information Table S1). The clean data was then successfully mapped to the reference genome and exhibited that mapping of reads ranged from 67.89 to 74.74% (Supplementary Information Table S1).

### Dynamics of DEGs in response to destruxin A

**Figure 1:**
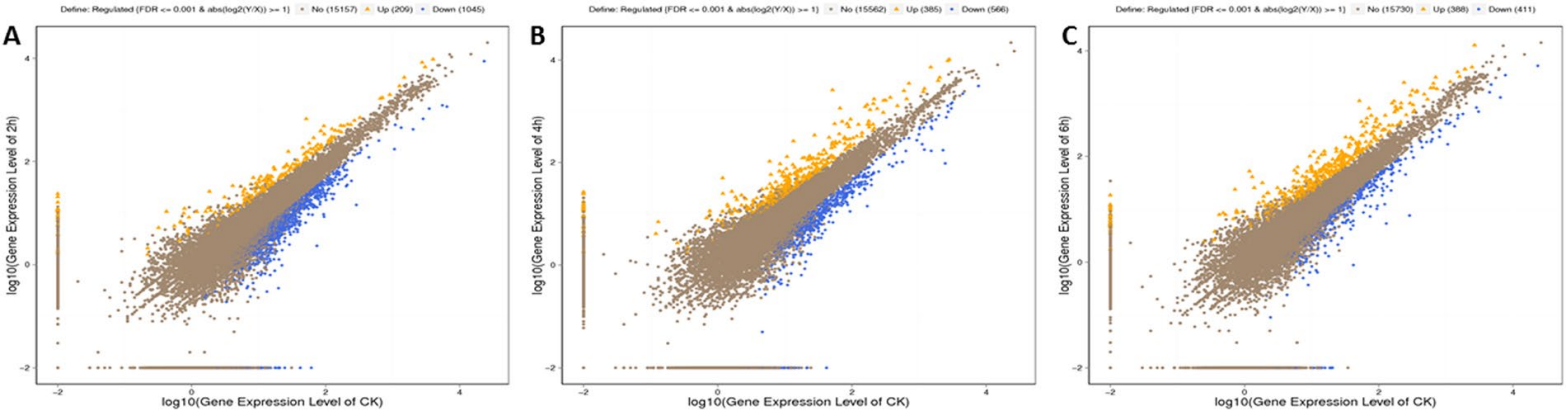
Scatter plot of all expressed genes in *Plutella xylostella* at 2 h, 4 h, and 6 h post-injection. X-axis and Y-axis present log2 value of gene expression. Blue means down-regulated genes, orange means up-regulated genes, and brown means non-regulated gene. (**A**) 2 h post-injection, (**B**) 4 h post-injection, (**C**) 6 h post-injection.

To explore the changes in the gene expression of *P. xylostella* larvae injected with destruxin A, the pairwise comparison was carried out between libraries to determine the DEGs. The screening threshold for genes relative to the control was set as the genes having a change of greater than 1-fold and FDR value less than 0.001 were scrutinized as DEGs. According to the results, compared to the control, there were 1254 (209 up-regulated and 1045 down-regulated), 951 (385 up- and 566 down-regulated), and 799 (388 up- and 411 down-regulated) genes that were significantly changed in *P. xylostella* after 2 h, 4 h, and 6 h, respectively (Fig. 1).

**Figure 2:**
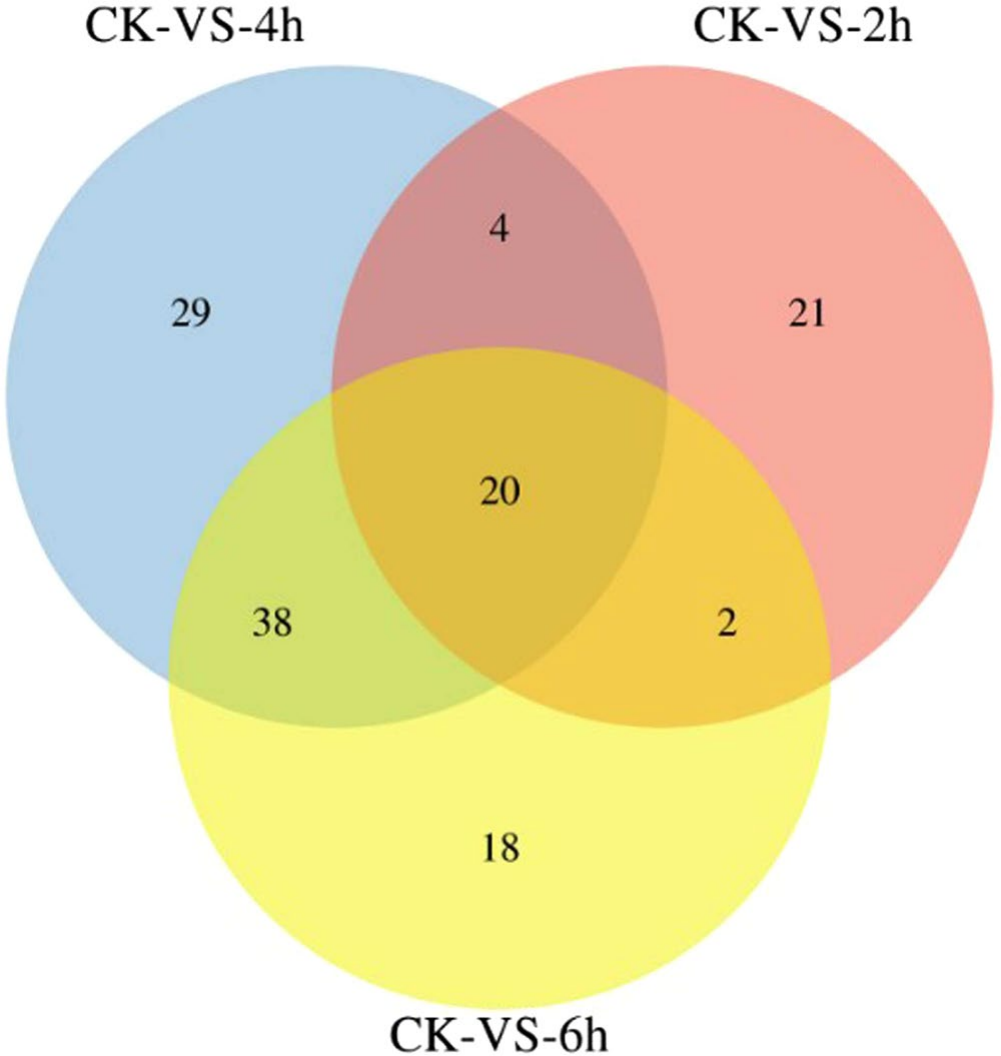
A Venn diagram of immunity-related differentially expressed genes in *P. xylostella* at 2 h, 4 h, and 6 h post-injection. The numbers in each circle show immunity-related differentially expressed genes in each comparison treatment and the overlapping regions display genes that are commonly expressed among the comparison treatments.

A Venn diagram analysis was performed to indicate the number of common and exclusive immunity-related DEGs among the three treatments (Fig. 2). There were 20 DEGs that were commonly expressed among all the treatments, while 4, 2, and 38 DEGs were commonly expressed among 2 h and 4 h, 2 h and 6 h, and 4 h and 6 h, respectively. Moreover, 21, 29, and 18 DEGs were specifically expressed in 2 h, 4 h, and 6 h, respectively (Fig. 2).

### Identification, expression pattern, and dynamics of immunity-related genes in response to destruxin A

A comprehensive analysis was performed to identify immunity-related genes in response to destruxin A in *P. xylostella* 4^th^ instar larvae by searching the genome and by combining BLAST search and GO annotation results. To increase the reliability of results, genes annotated as hypothetical or unknown proteins and genes with FPKM and fold change < 1 were filtered out. Finally, in total, 129 immunity-related genes were identified and categorized into different groups, such as signal recognition, signal modulation, signal transduction, effectors, and others (Supplementary Information Table S2).

**Figure 3:**
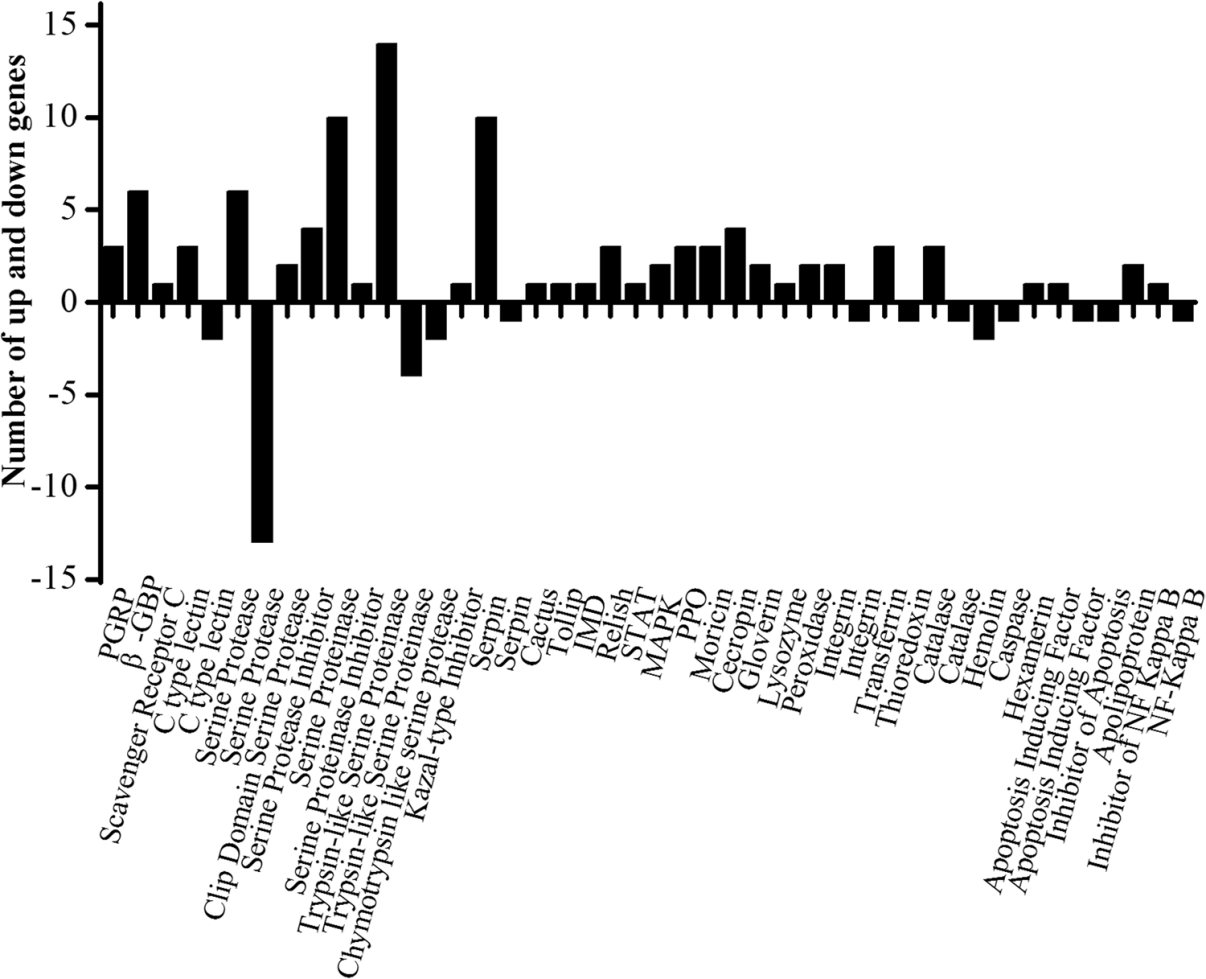
Functional classification of immunity-related DEGs in response to destruxin A.

**Figure 4:**
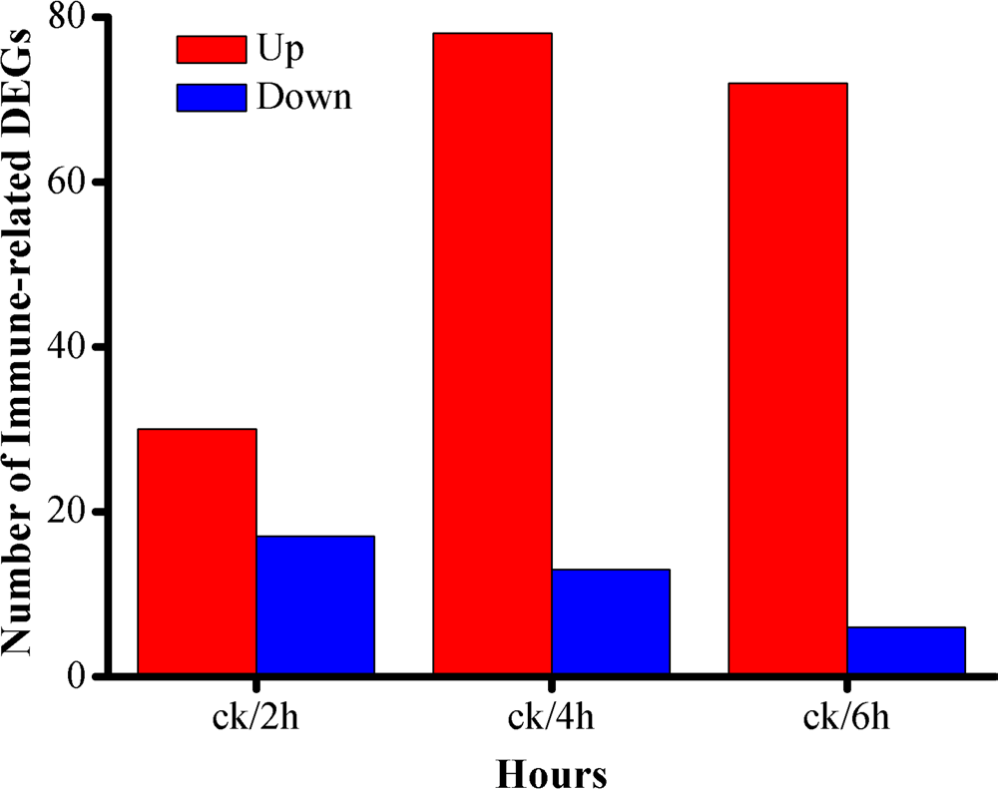
Screening of immunity-related DEGs in response to destruxin A at 2 h, 4 h, and 6 h post-injection.

**Figure 5:**
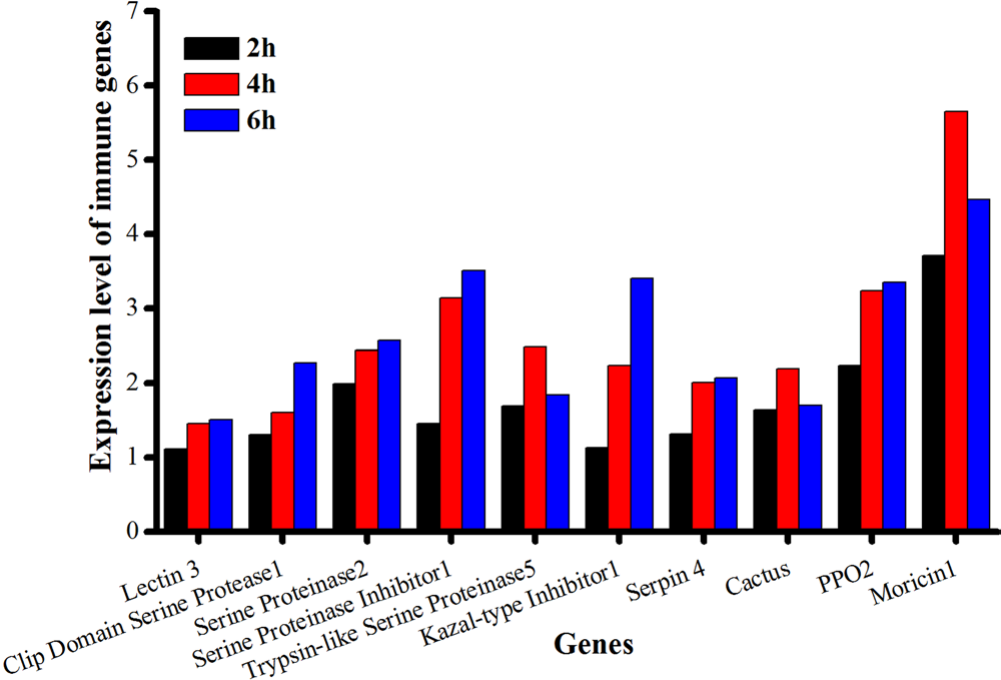
Screening of top ten immunity-related DEGs persistently expressed in response to destruxin A at 2 h, 4 h, and 6 h post-injection.

The immunity-related DEGs exhibited significant changes in the level of gene expression in response to destruxin A at different time courses (Figs 3 and 4). In the signal recognition group, PGRPs, βGRPs, and scavenger receptors were up-regulated in response to destruxin A. Whereas, 3 lectins were up- and 2 were down-regulated with lectin3 (px-105394158) showing persistently up-regulated expression with 1.11-fold, 1.45-fold, and 1.51 fold at 2 h, 4 h, and 6 h post-injection while lectin4 (105383689) down-regulated up to −9.07-fold at 2 h post-injection, respectively (Fig. 5 and supplementary information Table S2).

The genes included in signal modulation group, like serine protease, serine proteinase, and trypsin-like serine proteinase were observed to show up- or down-regulated expression pattern with 6, 10, and 14 up-regulated and 13, 0, and 4 down-regulated, respectively (Fig. 3). Among signal modulation genes, few genes were persistently expressed, including clip domain serine protease, serine proteinase, serine proteinase inhibitor, trypsin-like serine proteinase, kazal- type inhibitor, and serpin at 2 h, 4 h, and 6 h treatment, respectively (Fig. 5).

In the signal transduction group, cactus, toll, IMD, relish, STAT, and MAPK were up- regulated in response to destruxin A (Fig. 3). Among them, cactus showed persistent expression with 1.64-fold, 2.19-fold, and 1.69-fold change at 2 h, 4 h, and 6 h post-injection, respectively (Fig. 5).

The immune effector genes, including PPO, moricin, gloverin, lysozyme, and cecropin exhibited up-regulated expression pattern in response to destruxin A (Fig. 3). Among them, PPOs and moricins were persistently expressed with PPO2 (Px-105393465) showing up-regulated expression with 2.23-fold, 3.23-fold, and 3.34-fold and moricin1 (Px-105392533) 3.70-fold, 5.65-fold, and 4.46-fold change at 2 h, 4 h, and 6 h post-injection, respectively (Fig. 5).

The gene expression changes of *P. xylostella* larvae injected with destruxin A were explored by the pairwise comparison between libraries to determine the immunity-related DEGs. The screening threshold was same as described earlier. According to the results, compared to the control, there were 47 (30 up- and 17 down-regulated), 91 (78 up- and 13 down-regulated), and 78 (72 up- and 6 down-regulated) immunity-related genes that were significantly changed in *P. xylostella* after 2 h, 4 h, and 6 h, respectively (Fig. 4).

### Functional annotation of immunity-related genes in response to destruxin A

**Figure 6:**
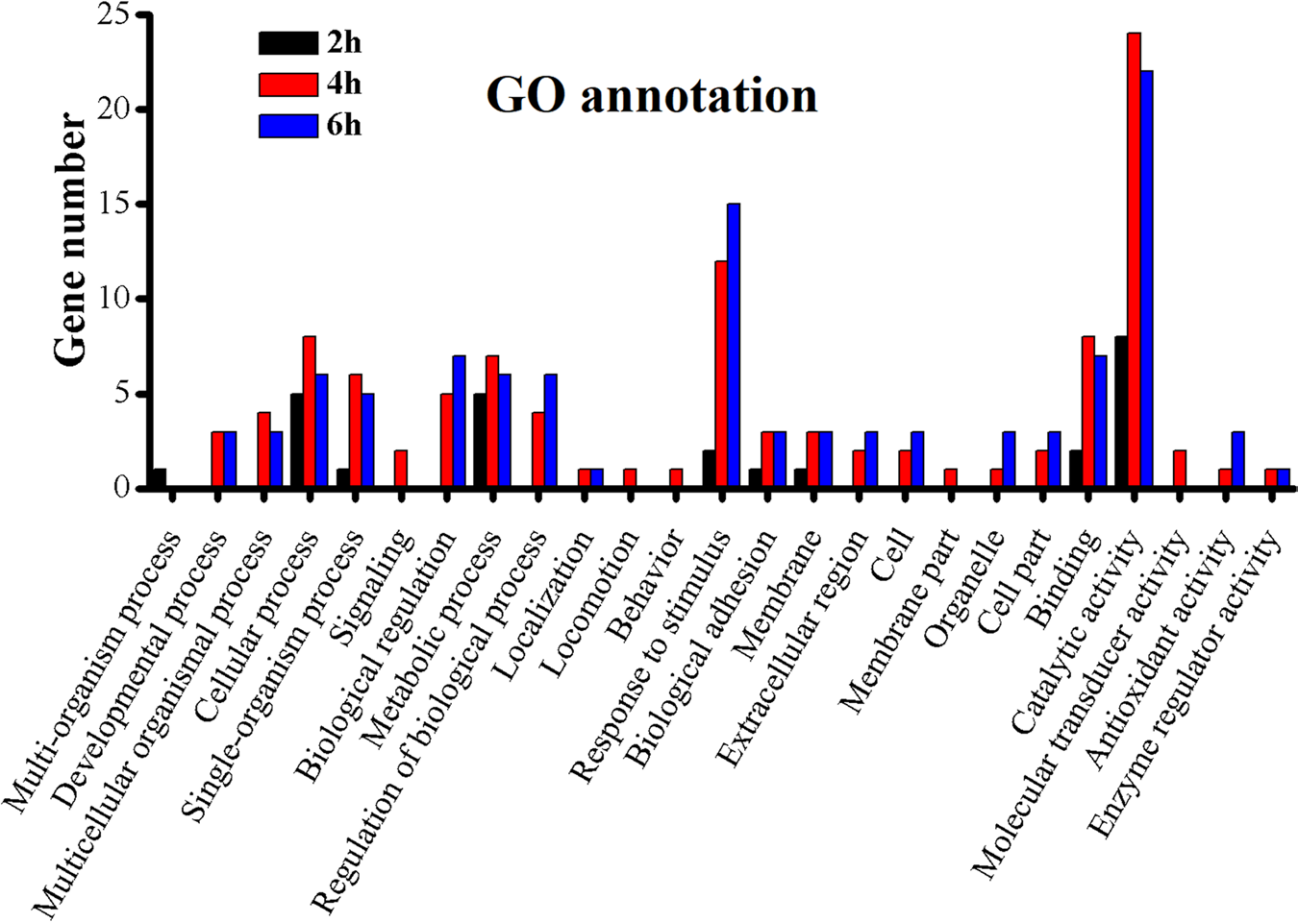
Summary of Gene ontology annotation. Functional classification of immunity- related DEGs at 2 h, 4 h, and 6 h post-infection in *P. xylostella* using gene ontology terms.

**Figure 7:**
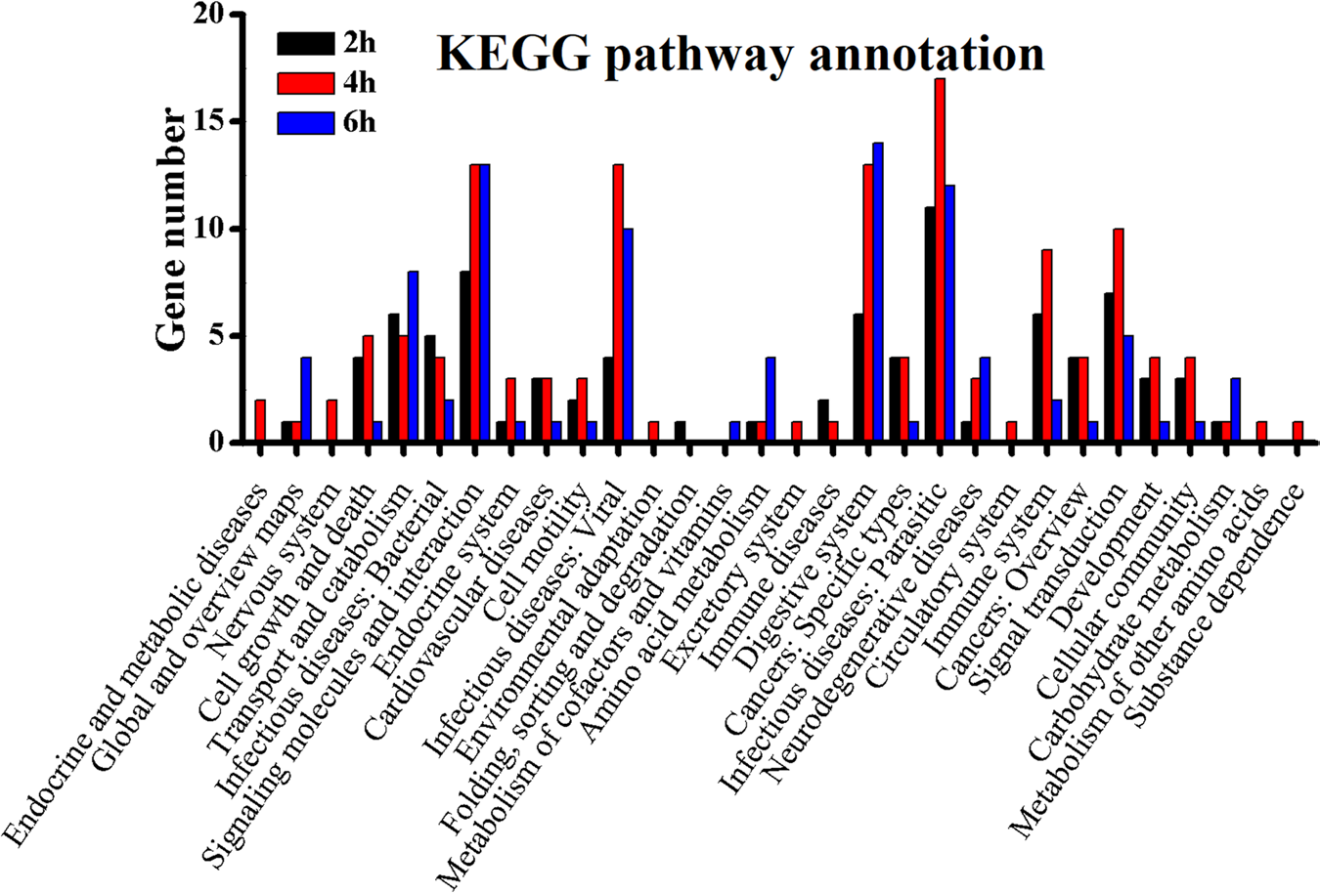
KEGG pathway annotation classification of immunity-related genes in *P. xylostella* infected with destruxin A at 2 h, 4 h, and 6 h. The abscissa is the KEGG classification, and the ordinate left is the gene number.

The Gene Ontology (GO) enrichment analysis and Kyoto Encyclopedia of Genes and Genomes (KEGG) analysis were carried out to gain knowledge of the potential function of immunity- related DEGs. In the gene repertoire of 2 h, 4 h, and 6 h, response to stimulus (2, 12, and 15); membrane (1, 3, and 3); and catalytic activity (8, 24, and 22) were most enriched in the categories of biological process, cellular component, and molecular function, respectively (Fig. 6). The KEGG classification system categorized immunity-related genes into different groups. In the gene repertoire of 2 h, 4 h, and 6 h, the top five enriched groups among KEGG categories included infectious diseases (viral), signaling molecules and interaction, digestive system, infectious diseases (parasitic), and signal transduction (Fig. 7).

### Validation of DEGs by RT-qPCR

**Figure 8:**
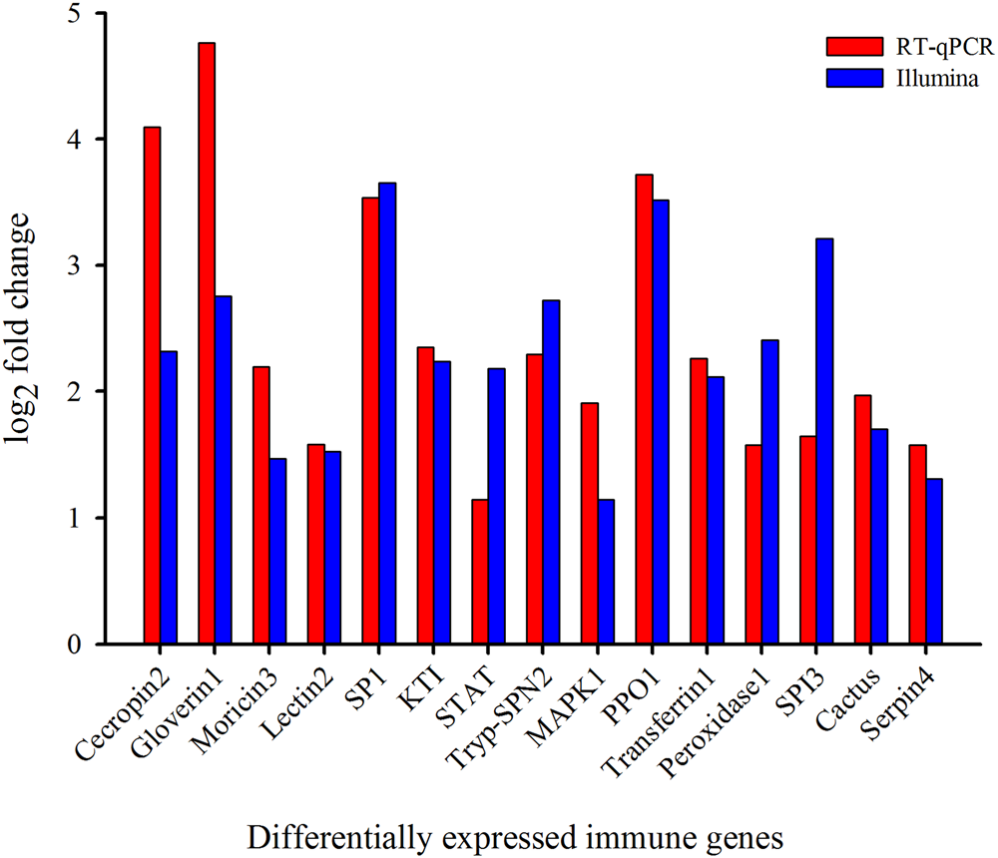
Validation of differential expression ratio (log2) achieved by RT-qPCR and RNA-Seq for immunity-related genes. Cecropin2, Cecropin (Px_105394859); Gloverin1, Gloverin (Px_105389810); Moricin3, Moricin (Px_105392532); Lectin2, Lectin (Px_105392416); SP1, Serine Protease (Px_105381787); KTI, Kazal-type Inhibitor (Px_105382984); STAT, STAT (Px_105396563); Tryp-SPN2, Trypsin-like Serine Proteinase (Px_105393249); MAPK1, Mitogen-activated Protein Kinase (Px_105380044); PPO1, Prophenoloxidase (Px_105393828); Transferrin1, Transferrin (Px_105384728); Peroxidase1, Peroxidase (Px_105388497); SPI3, Serine Protease Inhibitor (Px_105389206); Cactus, Cactus (Px_105384975); Serpin4, Serpin (Px_105396589).

**Figure 9:**
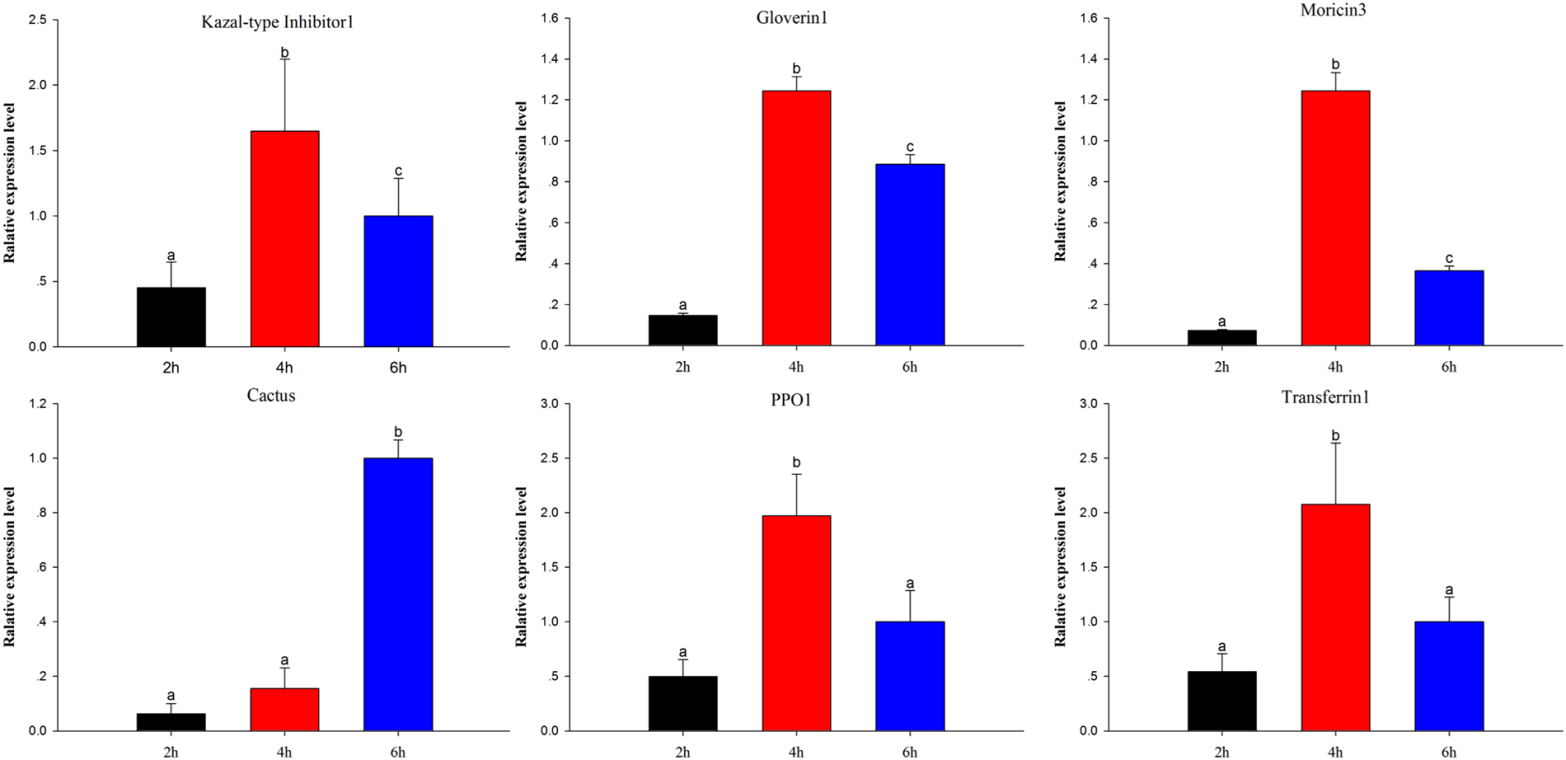
Expression of differentially expressed immunity-related genes at different time courses after destruxin A injection. Each vertical bar represents the mean ± SEM (*n* = 3) for various time points. Statistically significant differences in different groups are indicated by different letters at *P* < 0.05.

To validate DEGs results, 15 randomly selected immunity-related differentially expressed genes were analyzed by RT-qPCR (Fig. 8). In addition, 6 immunity-related genes were selected for further confirmation of results at 2 h, 4 h, and 6 h time courses (Fig. 9). The results exhibited that the trend of expression level for all the selected genes was in consistence to that of RNA-Seq.

## Discussion

The entomopathogenic fungi are considered as an environmentally friendly approach for the control of insect pests. Although, many species of entomopathogenic fungi exist in nature, however, *M. anisopliae* and *B. bassiana* have received more attention due to wide host range and capability of mass production^37^. During pathogenesis, these entomopathogenic fungi secrete virulence factors to accelerate the death of infected host^23^. Destruxins, the virulence factors of fungi, have been reported to exhibit high toxicity to various insect species when ingested or injected^12, 14, 38, 39^. Considering the importance of destruxins, we performed a genomic analysis of immune response of *P. xylostella* following injection of destruxin A at three different time points using high-throughput sequencing Illumina.

### Recognition of Microbes

In invertebrates, the initial line defense and self-protection system against invading microorganisms are known as innate immunity. The innate immune response begins with the recognition of pathogens mediated by pattern recognition molecules^40^. A number of pattern recognition receptors have been reported such as peptidoglycan recognition protein (PGRP), β-1,3-glucan recognition protein (βGRPs), galectins, C-type lectins (CTLs), and scavenger receptors (SCRs)^41^. The members of PGRP group have the ability to discriminate different types of invading bacteria and contain a C-terminal PGRP domain that is similar to a bacteriophage^42^. PGRPs can further be classified as short and long PGRPs depending on their length and existence of transmembrane domain. Some PGRPs were previously found to be down-regulated by destruxin A injection in *D. melanogaster* ^23^. Recently, it has been reported that PGRPs were up-regulated in *B. mori* hemocytes after injection of destruxin A^24^. In the present study, 3 PGRPs were identified and all three were up-regulated with PGRPS1 (px-105387866) up-regulated from 1.64-fold to 2.49-fold in response to destruxin A injection after 4 h and 6 h treatment (Supplementary Information Table S2). Our results exhibit that PGRPs activated the innate immune response of *P. xylostella* to destruxin A at the initial stage. Our study, in accordance with the previous study^24^ on *B. mori*, suggests that PGRPs may act as common protein recognition receptors (PRRs) regulating the immune response of insects against destruxin A.

The βGBPs are another PRRs family found in several insects^43–46^. In βGBPs, the presence of β-1, 3-glucanase-like domain is the characteristic feature of this family for detection of microbes. Previously, it has been reported that βGBPs were up-regulated by fungal infection in *Locusta migratoria* ^47, 48^. In consistence with these reports, here, we identified 6 βGBPs and all of them were up-regulated in response to the secondary metabolite of fungi, destruxin A, at 4 h and 6 h post-injection (Supplementary Information Table S2).

Scavenger receptors (SCRs), glycoproteins, are categorized into almost eight subfamilies with A, B, and C known as major subfamilies^49^. These different classes of SCRs are involved in different functions like phagocytosis of pathogens^50^ and apoptotic cell binding^51^. SCRs exhibited an up-regulated expression after the injection of destruxin A in *D. melanogaster* ^23^, similarly, in our study, we found only one SCR belonging to class C and it was also up-regulated after the injection of destruxin A (Supplementary Information Table S2). Recently, SCRs showed no change in expression in *B. mori* after destruxin A injection^24^. The reason may be the activation of different immune cell response to destruxin A in different insect species.

C-type lectins (CTLs), a carbohydrate binding proteins group, have been reported to play a vital role in the immune response of invertebrates, such as activation of PPO^52^, clearance of microbes^53^, and nodule formation mediated by hemocytes^54^. In our study, 5 CTLs were identified and 3 of them were up-regulated and 2 were down-regulated after destruxin A injection (Supplementary Information Table S2). In accordance with our results, C-type lectins were also found up- and down-regulated in *L. migratoria* in response to fungal infection^47^ whereas, no change in the expression of C-type lectins was observed in *B. mori* in response to destruxin A^24^.

### Signal Modulation

Prophenoloxidases (PPOs) are present in the form of inactive zymogen in the hemolymph. Soon after binding with PRRs, the PPOs are activated by an eventual cleavage of proteases^55^. The phenoloxidase, an important enzyme in the biosynthesis of melanin, catalyze the oxygenation of phenols to quinones and finally polymerizes into melanin^56^. It has been reported that besides mosquitoes, most of the insects contain 1-3 PPOs^46^. Here, we identified 3 PPO genes and all of these were up-regulated with PPO2 (px-105393465) persistently up-regulated in response to destruxin injection at different time courses (Supplementary Information Table S2). Similar to our results, PPO was up-regulated and its level was increased in *Schistocerca gregaria* and *Dialeurodes citri* after the infection of *M. anisopliae* and *Lecanicillium attenuatum*, respectively^57, 58^. These results suggest that PPOs exhibit more active response to pathogenic fungi and their secondary metabolites like destruxin A.

Serine proteases are known as the largest family of protein in insects and play an important role in different physiological processes, including digestion, development, and defense responses^59^. In our study, 51 serine proteases were identified, in total, and were up- and down-regulated in response to destruxin A at different time courses (Supplementary Information Table S2). A similar phenomenon of serine proteases was observed in *D. melanogaster* with up-and down-regulated expression in response to destruxin A^23^ suggesting that serine protease family was the most effected family by destruxin A in both *D. melanogaster* and *P. xylostella*.

Serine protease inhibitors (serpins), a super-family of proteins, are widely distributed among animals, plants, viruses, and bacteria^60^. Generally, they are almost 350-400 amino acid residues in length and can be found intra or extracellularly. Although the similarity of amino acid sequence ranges from 17 to 95% among all serpins, key conserved residues help in the inhibitory serpins folding into a metastable conformation particularly consisting of three β-sheets, eight to nine α -helices, and the solvent-exposed reactive center loop (RCL). The RCL of these SPIs binds to the particular target proteinase active site that is identical to substrate binding. When the cleavage of the serpin takes place at scissile bond (designated P1-P19), it goes through an important conformational transition, trapping the target proteinase covalently^61, 62^. Here, we identified 15 serine protease inhibitors in the genome of *P. xylostella* and most of these were up-regulated with serpin4 (px-105396589) consistently expressed in response to destruxin A at all three time courses (Fig. 5 and Supplementary Information Table S2). Our results suggest that the induction of serine protease inhibitors by destruxin A in *P. xylostella* may be to suppress the PPO system.

### Immune signaling pathways

The signal transduction pathways are triggered immediately after the recognition and modulation of invading microorganisms to produce antimicrobial compounds. Until now, four signal pathways related to insect immune system have been reported, including the Toll, Imd, JNK, and JAK/STAT^63^. Toll and Imd pathways play a vital role in microbes sensing. The Toll pathway combats fungi and Gram-positive bacteria, whereas, the Imd pathway of insects is involved only in the Gram-negative bacteria^64^. Previously, the Toll pathway was induced in response to destruxin A in *D. melanogaster* and *B. mori* ^23, 24^, here, in our study, the toll was also up-regulated after the injection of destruxin A. In the Imd pathway, here, we only identified Imd and relish showing up-regulated expression in response to destruxin A (Supplementary Information Table S2). The JNK and JAK/STAT pathways also play a vital role in response to pathogens alongside the Toll and Imd pathways. We identified one STAT gene that was induced in response to destruxin A at 4 h post-injection (Supplementary Information Table S2).

### Effectors

The effector genes are expressed following recognition of microbes, signal modulation, and transduction immune processes. The antimicrobial peptides production, phenoloxidase mediated melanization, and cellular apoptosis are triggered by these effectors. The antimicrobial peptides are induced in particular tissues, including fat bodies and hemocytes. Previously, it has been reported that antimicrobial peptides like cecropin, attacin, diptericin, and Metchnikowin were down-regulated in response to destruxin A in *D. melanogaster* ^23^ leading to a conclusion that destruxin has the capability to suppress the humoral immune response of *D. melanogaster*. Contrary to the above mentioned report, here, we identified that antimicrobial peptides, including moricin, cecropin, gloverin, and lysozyme were up-regulated in response to destruxin A, indicating that destruxin A was unable to suppress the immune response of *P. xylostella*. The reason may be that *P. xylostella* has strong ability to resist and limit the infection of destruxin as different insects have a different immune response to pathogens.

In conclusion, the present study adopted RNA-Seq and DGE analysis to identify differentially expressed genes, especially focusing on key immunity-related genes, after treatment with a mycotoxin, destruxin A. The activity of antimicrobial peptides was increased after treatment indicating that destruxin A was unable to directly suppress the antimicrobial peptides of *P. xylostella*. However, a series of functional validation experiments are needed to be performed to evaluate the immunity-related genes identified in the present study.

## Methods

### Insect strain, rearing, and preparation of destruxin A

A susceptible strain of *P. xylostella* was obtained from the Engineering Research Centre of Biological Control, Ministry of Education, South China Agricultural University, China and was not exposed to insecticides for 10 generations. The insects were reared at 25 ± 1 °C with 65% relative humidity under 14:10 h (light: dark) photoperiod. The entomopathogenic fungus *M. anisopliae* strain MaQ10 was used to isolate and purify destruxin A^65^ and its purity was assessed by high-performance liquid chromatography (HPLC). Finally, phosphate buffered saline (PBS, PH 7.4) was used to dilute destruxin A.

### Injection of destruxin A into *P. xylostella* larvae and RNA sample preparation

Firstly, the stock solution of destruxin A (200 μg/mL) was prepared and then 2 μL of that solution was injected to 4^th^ instar larvae of the susceptible strain of *P. xylostella*. The control larvae were treated with PBS. After treatment (2 h, 4 h, and 6 h post-injection), thirty larvae from each treatment and control were collected and then instantaneously frozen in liquid nitrogen. Total RNA was isolated using Trizol Total RNA Isolation Kit (Takara, Japan) following manufacturer’s instructions. The concentrations of RNA were assessed using Nanodrop (Bio-Rad, USA) and its integrity was determined on Agilent 2100 Bioanalyzer (Agilent, USA).

### cDNA library preparation and Illumina sequencing

Four DGE libraries (2 h, 4 h, 6 h, and control) were prepared using the Illumina Gene Expression Sample Prep Kit (Illumina, San Diego, CA). Briefly, ten μg of total RNA extracted from each treatment and control was used to isolate poly (A)^+^ mRNA using oligo (dT) magnetic beads. Fragmentation buffer was mixed with poly (A)^+^ mRNA and mRNA was digested into short fragments. First-strand and second-strand cDNAs were synthesized using random hexamers and RNase H and DNA polymerase I, respectively. The double strand cDNA was purified with magnetic beads. Finally, fragments were ligated with sequencing adaptors enriched by PCR amplification. During the QC step, Agilent 2100 Bioanalyzer and ABI Step One Plus Real-Time PCR System were used to qualify and quantify the sample libraries and then sequenced on the Illumina HiSeq^TM^ 2000 system (Illumina, USA). Illumina sequencing was performed at the Beijing Genomics Institute (BGI-Shenzhen, China).

### Mapping DEGs to *P. xylostella* genome

The raw reads were processed to create clean reads by the following methodology. Firstly, raw reads having adopters and unknown bases (>10%) were removed. Secondly, low quality reads having more than 50% of bases with a quality value less than 5 were filtered out. The high-quality clean tags were mapped to reference sequences using Bowtie^66^ and to reference genome using HISAT^67^. To quantify the gene expression level, RSEM analysis^68^ was carried out in order to acquire read count of each gene of each sample, based on the mapping results. Finally, the gene expression level was calculated using fragments per kilobase per million (FPKM) method^69^.

### Functional analysis of differentially expressed genes

Differential expression analysis between treatment and control conditions was implemented using a rigorous algorithm. The threshold of P-value was determined using the false discovery rate (FDR) method in multiple tests^70^ and an FDR value of < 0.001 and the absolute value of log2 ratio ≥ 1 was set as a threshold to find out genes with significant differential expression. Hierarchical cluster analysis was carried out with cluster^71^ and Java Treeview softwares^72^.

The genome of *P. xylostella* (GCA_000330985.1) was used as the background to determine GO terms enriched within the DEG dataset using hypergeometric test and a corrected P-value (≤0.05) as a threshold in order to find out significantly enriched terms. Finally, Kyoto Encyclopedia of Genes and Genomes (KEGG) pathway enrichment analysis was performed to identify significantly enriched pathways within the DEG datasets compared with the genome database using hypergeometric test and a corrected P-value (≤0.05) as a threshold.

### Validation of DEGs results by RT-qPCR

Real-time quantitative PCR was performed to confirm the expression levels of mRNA displayed by Illumina sequencing results and 15 immunity-related DEGs were selected from the comparison of control vs. treatments. In addition, 6 immunity-related DEGs were selected for further confirmation of results from all three time (2 h, 4 h, and 6 h) courses. Total RNA was isolated from each sample as mentioned earlier. The RNA sample (1 μg) was treated with DNaseI (Fermentas, Glen Burnie, MD, USA) following manufacturer’s protocol and then complementary DNA was synthesized using M-MLV reverse transcriptase (Promega, USA). The RT-qPCR was carried out on a Bio-Rad iQ2 optical system (Bi-Rad) using SsoFast EvaGreen Supermix (Bio-Rad, Hercules, CA, USA) following the manufacturer’s guidelines. The amplification cycling parameters were: 95 °C for 30 s, 40 cycles of 95 °C for 5 s, and 55 °C for 10 s with a dissociation curve generated from 65–95 °C to ensure the purity of PCR products^73^. The ribosomal protein S13 (RPS13) was used as an internal control for normalization^74^ and the relative expression of genes was calculated using the 2^−ΔΔCT^ method^75^. All the experiments were performed with three independent biological repeats. The primer sequences used in RT-qPCR are presented in Supplementary Information Table S3.

## Electronic supplementary material

Supplementary Information
