# Supplementary material for: Identification of immunity-related genes in Plutella xylostella in response to fungal peptide destruxin A: RNA-Seq and DGE analysis
Source: Sci Rep. 2017 Sep 8;7:10966. doi: 10.1038/s41598-017-11298-7 (PMC5591186; doi:10.1038/s41598-017-11298-7)
Supplement: Supplementary file 1 — Supplementary Information [file 41598_2017_11298_MOESM1_ESM.pdf]

Identification of immunity-related genes in *Plutella xylostella* in response to fungal peptide destruxin A: RNA-Seq and DGE analysis

Muhammad Shakeel[†1], Xiaoxia Xu[†1], Jin Xu[1], Xun Zhu[2], Shuzhong Li[1], Xianqiang Zhou[3], Jialin Yu[3], Xiaojing Xu[3], Qiongbo Hu[1], Xiaoqiang Yu[4] and Fengliang Jin[1*]

[1]College of Agriculture, South China Agricultural University, Laboratory of Bio-Pesticide Creation and Application of Guangdong Province, Guangzhou, P. R. China.

[2] State Key Laboratory for Biology of Plant Disease and Insect Pests, Institute of Plant Protection, Chinese Academy of Agricultural Sciences, Beijing 100193, China

[3]BGI-Shenzhen, Shenzhen, P. R. China.

[4]School of Biological Sciences, University of Missouri-Kansas City, Kansas City, MO 64110, USA

Correspondence: Fengliang Jin, Laboratory of Bio-Pesticide Creation and Application of Guangdong Province, College of Agriculture, South China Agricultural University, Guangzhou 510642, China Tel.: +86 2085280203; fax: +86 20 85280293.

E-mail: jflbang@scau.edu.cn

[†] These authors contributed equally to this work.

Supplementary information Table 1. Statistics of DGE sequencing

Supplementary information Table 2. Immunity-related genes identified in *P. xylostella* genome in response of destruxin A

Supplementary information Table 3. Primers used for RT-qPCR in the present study

Table S1 Statistics of DGE sequencing

Sample	Raw Reads	Clean Reads	Q20 (%)	Q30 (%)	GC Content (%)	Total Mapped (% of Clean Data)
**2h**	7,098,592	7,098,592	98.8	96.7	49.52	74.74
**4h**	7,439,232	7,439,232	98.2	94.4	47.49	67.89
**6h**	7,252,990	7,252,990	98.9	96.8	48.75	74.72
**Control**	7,061,594	7,061,594	98.2	94.6	47.8	67.89

Table S2. Immunity-related genes identified in *P. xylostella* genome in response of destruxin

A

Gene Name	Gene ID	Gene Length	Protein Length	Accession No:	Min: E-value	Identity
**Recognition**						
**PGRP**						
Px_PGRP1	105387866	815	206	AFV15800.1	2.82E-60	60.23
Px_PGRP2	105386207	761	205	AFV15800.1	6.50E-61	60.8
Px_PGRP3	105391041	690	195	BAF36823.1	4.98E-91	87.1
**β-GBP**						
Px_β-GBP1	105389999	1577	490	Q8MU95.1	0.00E+00	65.91
Px_β-GBP2	105397355	1429	428	Q8MU95.1	0.00E+00	66.95
Px_β-GBP3	105390012	1655	429	AFK24449.1	0.00E+00	85.15
Px_β-GBP4	105397356	1822	479	AFK24449.1	0.00E+00	98.75
Px_β-GBP5	105380183	1618	458	AFK24449.1	0.00E+00	95.39
Px_β-GBP6	105390014	1494	489	Q8MU95.1	1.26E-128	49.06
**Scavenger Receptor**						
Px_ScR1	105386497	1985	592	ABB92836.1	1.19E-111	50.25
**Lectin**						
Px_Lectin1	105380860	546	179	AIR96002.1	1.98E-41	53.28
Px_Lectin2	105392416	1268	223	NP_001091747.1	6.62E-115	84.21
Px_Lectin3	105394158	1373.89	322	AIR95999.1	2.67E-127	65.3
Px_Lectin4	105383689	1290	307	AFC35299.1	7.98E-89	52.12
Px_Lectin5	105398161	1810	578	EHJ77925.1	8.12E-112	43.03
**Modulation**						
**Serine Protease**						
Px_SP1	105381787	1012	269	EHJ74628.1	7.17E-12	29.69
Px_SP2	105380881	591	128	XP_004922188.1	8.54E-10	42.11
Px_SP3	105380609	1544	416	XP_004922188.1	6.38E-107	51.3
Px_SP4	105390133	1276	351	EHJ75838.1	2.91E-67	44.11
Px_SP5	105397691	751	233	ACD44927.1	2.35E-31	35.09
Px_SP6	105380869	827	252	AGR92345.1	1.80E-94	68.07
Px_SP7	105388354	557	143	BAH58096.1	1.24E-09	31.3
Px_SP8	105391008	1030	289	ACR15987.2	1.67E-56	42.91
Px_SP9	105388737	874	196	XP_008203180.1	1.86E-07	38.96
Px_SP10	105394363	688	200	ADT80832.1	4.25E-26	37.5
Px_SP11	105392198	880	265	AGR92347.1	6.79E-58	46.09

Px_SP12	105388678	850	260	AGR92345.1	3.59E-77	55.38
Px_SP13	105393231	930	293	EHJ74828.1	2.58E-34	35.71
Px_SP14	105394388	1276	351	EHJ75838.1	2.91E-67	44.11
Px_SP15	105395982	1734	549	XP_004932654.1	9.88E-51	72.97
Px_SP16	105398015	1565	467	XP_004929850.1	4.87E-179	62.05
Px_SP17	105389290	1387.35	379	XP_004928024.1	5.76E-101	53.03
Px_SP18	105380905	5328	1550	EHJ71121.1	0.00E+00	60.74
Px_SP19	105388738	659	143	BAH58096.1	2.47E-09	30
**Clip Domain Serine Protease**						
Px_cSP1	105395144	2534	383	NP_001036891.1	2.22E-129	61.25
Px_cSP2	105386393	2400.75	730	NP_001036891.1	1.73E-129	61.25
**Serine Protease Inhibitor**						
Px_SPI1	105383414	2262	641	AEW46893.2	6.74E-90	46.38
Px_SPI2	105390552	1683	397	NP_001037205.1	3.28E-136	60.2
Px_SPI3	105389206	1763	407	NP_001139706.1	2.48E-57	34.28
Px_SPI4	105387668	1427	410	NP_001139706.1	8.98E-60	35.57
**Serine Proteinase**						
Px_SPN1	105395634	1342	387	NP_001040462.1	4.17E-96	53.78
Px_SPN2	105391132	1467	390	NP_001040462.1	2.85E-125	58.53
Px_SPN3	105380684	1479	483	EHJ70457.1	2.56E-81	38.65
Px_SPN4	105380637	1705	464	ACI32835.1	1.89E-147	65.41
Px_SPN5	105394340	1744	467	ACI32835.1	1.35E-148	65.95
Px_SPN6	105394347	1615	450	EHJ70457.1	2.60E-82	41.12
Px_SPN7	105396596	1452	390	NP_001040462.1	9.67E-126	57.99
Px_SPN8	105384594	783.28	241	ACI45418.1	4.74E-25	37.6
Px_SPN9	105390003	2708	846	EHJ70705.1	1.02E-87	40.57
Px_SPN10	105396174	1874	484	AAR29602.1	1.66E-83	51.49
**Serine Proteinase Inhibitor**						
Px_SPNI1	105383822	884	156	AAQ22771.1	4.64E-14	40.4
**Trypsin-like Serine Proteinase**						
Px_Tryp-SPN1	105380873	2612	805	EHJ67268.1	3.69E-103	48.54
Px_Tryp-SPN2	105393249	1904	490	AFK93534.1	1.02E-120	50.75
Px_Tryp-SPN3	105397224	1673	290	AFK93534.1	3.87E-121	51.01
Px_Tryp-SPN4	105397463	1503	354	EHJ78263.1	3.86E-101	53.87
Px_Tryp-SPN5	105386435	1650	354	EHJ78263.1	2.12E-79	45.04
Px_Tryp-SPN6	105387360	1558	354	EHJ78263.1	7.65E-100	52.39

Px_Tryp-SPN7	105391595	1629	485	AFK93534.1	1.28E-137	50.72
Px_Tryp-SPN8	105386282	2100	657	AFK93534.1	2.77E-82	50.18
Px_Tryp-SPN9	105394089	1501	387	EHJ75901.1	2.35E-66	39.12
Px_Tryp-SPN10	105385437	1297	333	AAV91007.1	8.91E-88	50.9
Px_Tryp-SPN11	105397808	1154	325	AAV91007.1	4.44E-48	47.23
Px_Tryp-SPN12	105383936	1277	271	ADK66277.1	7.20E-50	42.63
Px_Tryp-SPN13	105392752	963	286	ADK66277.1	2.39E-46	39.63
Px_Tryp-SPN14	105383574	865	272	ADK66277.1	8.90E-47	40
Px_Tryp-SPN15	105383595	728	225	ADK66277.1	3.74E-55	46.64
Px_Tryp-SPN16	105385127	593	185	AEP25403.1	7.11E-65	71.88
Px_Tryp-SPN17	105392836	696	156	AIR09766.1	2.70E-44	61.87
Px_Tryp-SPN18	105385090	872	156	AIR09766.1	3.21E-44	61.87
**Chymotrypsin-like Serine Protease**						
Px_ChymTryp-SP1	105388849	1189	304	NP_001040430.1	3.11E-60	47.08
Px_ChymTryp-SP2	105380856	1023	289	ABR88231.1	4.78E-72	51.49
**Kazal-type Inhibitor**						
Px_KTI	105382984	802	190	ADF97836.1	1.57E-23	37.72
**Serpin**						
Px_Serpin1	105395080	1350.54	422	BAD52261.1	4	99.49
Px_Serpin2	105390553	1247	396	AGK24648.1	1.00E-173	98.1
Px_Serpin3	105383415	2892	897	AHN49717.1	0	98.75
Px_Serpin4	105396589	676	180	AHN49717.1	8.31E-96	98.89
Px_Serpin5	105397282	1994	450	BAF36821.1	0	99.56
Px_Serpin6	105396587	1659	450	BAF36821.1	0.00E+00	99.33
Px_Serpin7	105383394	1941	447	BAF36821.1	0	98.67
Px_Serpin8	105390551	2438	412	AGM20455.1	0.00E+00	99.76
Px_Serpin9	105393397	1800	444	AHN49718.1	0	93.92
Px_Serpin10	105392292	601	199	BAF36820.1	5.99E-06	55.81
Px_Serpin11	105386098	5485	1418	ACG61190.1	0	54.61
**Signaling Pathway**						
**Toll Pathway**						
Px_Cactus	105384975	2403	350	AHH80649.1	2.19E-70	44.48
Px_Tollip	105398186	1545	273	KDR16420.1	5.10E-80	56.54
**Imd Pathway**						
Px_Imd	105396764	1150	251	AFK75937.1	9.99E-117	84.46
**Relish**						
Px_Relish1	105386073	3220	955	AEO51739.1	0.00E+00	48.82
Px_Relish2	105393889	1772	524	AIA24469.1	3.42E-123	48.4

Px_Relish3	105391886	3165	954	AEO51739.1	0	48.71
**JAK/STAT**						
Px_STAT	105396563	1676	536	ABL63639.1	0	64.44
**MAPK Pathway**						
Px_MAPK1	105380044	2990	716	XP_004931740.1	0	66.3
Px_MAPK2	105390812	2283	221	NP_001036921.1	7.26E-108	95.41
**Effectors**						
**Prophenoloxidase**						
Px_PPO1	105393828	1559.98	405	BAF36824.1	0	70.37
Px_PPO2	105393465	2479	790	BAF36824.1	1.82E-144	92.28
Px_PPO3	105384195	2224	382	ADF43208.1	9.69E-150	63.19
**Moricin**						
Px_Moricin1	105392533	436	65	ABQ42576.1	1.05E-11	75
Px_Moricin2	105392531	434	65	ABQ42576.1	1.99E-10	76.32
Px_Moricin3	105392532	451	65	ABQ42576.1	2.03E-10	76.32
**Cecropin**						
Px_Cecropin1	105394860	510	65	BAF36816.1	1.03E-16	73.02
Px_Cecropin2	105394859	684	65	ADA13281.1	1.58E-17	73.85
Px_Cecropin3	105394858	512	65	ADA13281.1	2.06E-17	73.85
Px_Cecropin4	105397888	582	65	ADA13281.1	1.06E-17	73.85
**Gloverin**						
Px_Gloverin1	105389810	628	172	ACM69342.1	5.04E-54	60.57
Px_Gloverin2	105389803	489	128	ACM69342.1	1.93E-51	89.91
**Lysozyme**						
Px_Lysozyme1	105382813	548	140	EHJ67777.1	6.79E-50	71.54
**Others**						
**Peroxidase**						
Px_Peroxidase1	105388497	2079	627	BAM17900.1	1.32E-177	50.66
Px_Peroxidase2	105386582	2212	637	BAM17900.1	0	54.17
**Integrin**						
Px_Integrin1	105394193	2349	746	ACS66819.1	0	90.3
Px_Integrin2	105386408	2973	943	EHJ72232.1	0	49.26
Px_Integrin3	105397540	2397	740	AII79417.1	0.00E+00	57.64
**Transferrin**						
Px_Transferrin1	105384728	1904	534	BAF36818.1	0	96.89
Px_Transferrin2	105393952	1006	325	BAF36818.1	0	99.05
Px_Transferrin3	105386270	2553.03	656	XP_004926508.1	0	57.79
**Thioredoxin**						
Px_Thioredoxin	105382641	877	165	XP_004931380.1	8.24E-56	64.02
**Catalase**						
Px_Catalase1	105398438	1767	508	NP_001036912.1	0	82.09

Px_Catalase2	105389213	1429	474	XP_004924808.1	1.83E-145	53.4
Px_Catalase3	105390515	1686	508	NP_001036912.1	0	82.48
Px_Catalase4	105382676	2235	581	AFC98367.1	0	80.12
**Hemolin**						
Px_Hemolin1	105382056	1403	415	ACN69054.1	0	94.46
Px_Hemolin2	105394779	1451	415	ACN69054.1	0	94.46
**Caspase**						
Px_Caspase	105385382	2175	300	ADK24087.1	1.54E-176	99.67
**Hexamerin**						
Px_Hexamerin	105388113	2219	670	EHJ65896.1	3.09E-164	44.05
**Apoptosis Inducing Factor**						
Px_API1	105392107	1554	469	NP_001189462.1	1.15E-79	44.64
Px_API2	105386877	1914	602	XP_004926894.1	0	67.89
**Inhibitor of Apoptosis**						
Px_IAP	105385525	4118	437	NP_001189458.1	3.20E-75	39.58
**Apolipoprotein**						
Px_Apoliopprotein1	105381848	1170	214	XP_004923379.1	3.35E-51	44.17
Px_Apoliopprotein2	105392552	4170	1390	BAN58736.1	0	65.81
**Inhibitor of NF Kappa B**						
Px_INFKB	105396222	2569	769	XP_004928169.1	0	60.49
**NF-Kappa B**						
Px_NFKB	105390718	2296	739	XP_004924233.1	3.11E-175	54.69

Table S3 Primers used for RT-qPCR in the present study

Gene Name	Gene ID	Direction	Sequence (5'-3')
Px_Cecropin2	105394859	Forward	CAGGTGGAATCCGTTCAA
		Reverse	GAAGTGGCTTGTCCTATGA
Px_Gloverin1	105389810	Forward	GTTAGTCGCTTATGTGTCAG
		Reverse	CGTCGTTGAAGATGTCTTG
Px_Moricin3	105392532	Forward	GATTCTTCCACTTGCTGATG
		Reverse	CCTTCCGTATAACTCTTCCG
Px_Lectin2	105392416	Forward	CAGGATAAGGTGAAGTACATCT
		Reverse	CCGTCGTTGTAGAAGTTGT
Px_SP1	105381787	Forward	ACCACTTCGTCTCCTGAT
		Reverse	TTCTTCACAGCACCAGTC
Px_KTI	105382984	Forward	CGAGTGTTTGTTGAACTG
		Reverse	CAGTTGCTCCTCAGATAC
Px_MAPK1	105380044	Forward	GTGACTGAGAATAGAGATG
		Reverse	GTATCATAGTTCCTGTAGG
Px_PPO1	105393828	Forward	CCTGAAGACATACTGTAC
		Reverse	GTCACCATCTTCTGTATC
Px_Transferrin1	105384728	Forward	CCCTCTGTACTACCTTAT
		Reverse	CTCTAATTTGGCTACTCC
Px_Peroxidase1	105388497	Forward	GTCTCAGAACAGATACTAC
		Reverse	CTCGTACCAATGTCTATC
Px_SPI3	105389206	Forward	GGTAGTATCAGCAAGTTC
		Reverse	CTCTGTCTTCTTATCCAC
Px_Cactus	105384975	Forward	CTTAGTGAGGAAGATCCT
		Reverse	CATCACCTATAGTGACTG
Px_Serpin4	105396589	Forward	CCGAATAAAAGGGACTAC
		Reverse	GACTTTCCTGATCATCTG
Px_STAT	105396563	Forward	CGACACTGAGCTTATTAG
		Reverse	CCTGTTATCTGGTTAGTC
Px_Tryp-SPN2	105393249	Forward	GCAGACCTTGGTTATATC
		Reverse	GATGAAGCTCTTGTACTC
RPS13	Reference Gene	Forward	TCAGGCTTATTCTCGTCG
		Reverse	GCTGTGCTGGATTCGTAC
